# Menopause in working life

**DOI:** 10.5271/sjweh.4258

**Published:** 2025-11-01

**Authors:** Johanna Kausto, Chief Researcher

An increasing proportion of the working-age population in Western countries consists of middle-aged or older women. Among EU countries in 2024, the employment rate of women aged 55–64 was 59%. The rate was lowest in Romania, at 43%, and highest in Estonia, at 78% (1). In Finland in 2024, the employment rate for women aged 45–54 was 84%, and 73% for those aged 55–64 (2). In high-income countries, menopause typically begins at an average age of 50–51, with a range of 45–55 years (3, 4). Early menopause occurs between 40–44 years (5). Therefore, a growing number of women in many countries will experience menopause during their professional careers (6).

Menopause affects women’s lives in various ways. Symptoms such as hot flashes, sleep disturbances, mood swings, and fatigue are quite common and may impair work performance, concentration, and the ability to manage tasks (6–10). Additionally, menopause can cause physical symptoms such as joint and muscle pain (11), which may make working more difficult. At the same time, it is important to remember that menopause is not a disease but a natural part of aging (12, 13).

## Support for women facing menopause in the workplace

Supporting work ability for those experiencing menopause is an important aspect of gender and age equality in the workplace and overall occupational well-being. Recognizing the need for support has sparked societal discussions, and many countries have developed guidelines for employers and employees on menopause-related practices. These include policies on sick leave reporting, flexible working arrangements, information sharing, training, and workplace modifications. The role of occupational health care is important and tied to the prevailing social and healthcare system, national policies and available resources (14, 15).

## The need for research

In Finland, the impact of menopause on work ability has only recently entered public discussion. Recognizing menopause symptoms and providing effective measures to strengthen work ability and work participation has already been mentioned in government policy and current care guidelines (16), but funding for research and concrete measures are still not available. Addressing menopause overlaps with, but is not the same as, the broader issue of improving age management and supporting the careers of employees aged ≥55 (17).

Overall, menopause—as an aspect of women’s health and wellbeing—has received inadequate research attention and funding (18, 19). So far, research evidence on menopausal symptoms and work-related outcomes is mostly correlational and high-quality prospective studies are needed (10). Among the rare prospective cohort studies, one (20) suggested that women with severe menopausal symptoms had a higher risk of employment exit. Another prospective, register-based cohort study reported that an earlier menopausal transition predicted lower work participation and a higher disability pension rate in the following years (21).

The associations between the experiences of menopausal symptoms, the surrounding work context and work-related outcomes are complex, but—so far—the evidence is limited. For example, the experience of menopause and the impact of menopausal symptoms on work ability and work participation may differ by various background factors of the individuals and features of the cultural and social context. Also, menopausal symptoms may modify the relationships between physical and psychosocial work features and work ability and wellbeing (6, 22, 23). At present, there is a lack of adequate evidence to demonstrate the effectiveness of workplace measures for women dealing with menopausal symptoms (15, 24, 25). Qualitative research is important, as experiences of menopause are both individual (although often shared) and influenced by physical, social, and cultural contexts. Menopause can be a sensitive subject (26, 27). In low- and middle-income countries, the topic may be ignored due to negative perceptions, insufficient awareness, and societal stigma (28).

## Conclusion

In a rapidly ageing global workforce, menopause in working life is becoming increasingly important and topical. A multidisciplinary approach (26), involving efforts from disciplines such as medicine, public health, economics, psychology, sociology, and gender studies, is needed to comprehensively examine the working life of menopausal women and develop interventions to protect and improve their health, workability, and labor market participation.
